# Systematic Review of Teleneurology: Methodology

**DOI:** 10.3389/fneur.2012.00156

**Published:** 2012-11-08

**Authors:** Mark N. Rubin, Kay E. Wellik, Dwight D. Channer, Bart M. Demaerschalk

**Affiliations:** ^1^Department of Neurology, Mayo ClinicRochester, MN, USA; ^2^Department of Library Sciences, Mayo ClinicScottsdale, AZ, USA; ^3^Department of Neurology, Mayo ClinicPhoenix, AZ, USA

**Keywords:** teleneurology, systematic review, telemedicine, neurology, telecommunications, remote consultation

## Abstract

**Background:** The use of two-way audio-visual technology for delivery of acute stroke is supported by a well established literature base. The use of telemedicine for general neurologic consultation has been reported across most subspecialties within the field, but a comprehensive systematic review of these reports is lacking. **Purpose:** To conduct a systematic review of the published literature on teleneurologic consultation beyond stroke. **Data sources:** Databases Ovid MEDLINE, EMBASE, PsychINFO, CINAHL, and Cochrane were searched with keywords, “teleneurology,” and numerous synonyms and cross-referenced with neurology subspecialties. The search yielded 6,615 potentially eligible hits, which were independently reviewed by two investigators. Ultimately 375 unique studies met eligibility criteria and were included in the review. **Study selection:** Studies were included if the title or abstract expressed use of two-way AV communication for a clinical neurologic indication other than stroke. **Data extraction:** Each article was classified using a novel scoring rubric to assess the level of functionality, application, technology, and evaluative stage. **Data analysis:** Articles were hierarchized within a subspecialty category. Overall subspecialty scores were assigned based on aggregate of scores across papers in each category. **Conclusion:** Use of telemedicine for general and most subspecialty neurologic consultation, beyond stroke, appears very promising but the clinical science is nascent.

## Introduction

Telemedicine in neurology is utilized and has been studied primarily in the acute stroke setting (Meyer et al., 2008; Demaerschalk et al., 2012). The state of telestroke practice has matured to the point that there are specific American Heart Association/American Stroke Association statements detailing the evidence for its use (Schwamm et al., 2009b) and guidelines for its implementation into stroke systems of care (Schwamm et al., 2009a). Moreover, telestroke practice is at a stage where health economic analyses have been performed and suggest long-term cost effectiveness from the societal perspective (Nelson et al., 2011). Neither practice models nor a rigorous literature base exists for teleneurology beyond stroke. There are widespread reports of remote communication *via* various modalities [e.g., telephone (Hill et al., 2009), videophone (Vesmarovich et al., 1999), e-mail (Ahmed et al., 2010), two-way audio/visual (Theodoros et al., 2003)] to address various non-stroke neurologic issues in the literature but no systematic review thereof. The aim of the study group is to conduct a systematic review of the medical literature describing the use of two-way audio-visual (AV) communication in order to address general (e.g., non-stroke) neurologic indications, categorized by neurology subspecialty. This manuscript describes the methods of the systematic review and introduces a novel rubric for appraising, scoring, and hierarchizing teleneurological studies.

## Methods

In January 2011 Ovid MEDLINE was searched from 1948 forward to identify relevant studies for review. A search strategy utilizing MeSH (Medical Subject Headings), textwords, and telemedicine journal titles was conducted to create one large set to later combine with the specialized areas within the field of neurology. This basic set included the MeSH terms Telemedicine, Telecommunications, and Remote Consultation. Textwords included telestrokolog:, telestroke, teleneurolog:, telemedicine, telecare, telehealth, telerehabilitation, telediagnosis, remote monitoring, remote evaluation, and teleconsult. Search terms were truncated to include variant endings. Journals included were *Telemedicine & Telehealth Networks, Telemedicine & Virtual Reality, Telemedicine Journal, Telemedicine & eHealth, Telemedicine Today, and Journal of Telemedicine & Telecare*.

All MeSH, textwords, and journals were combined using the Boolean operator OR. The resulting set was limited to humans and the publication types “comment” and “letter” were removed. This basic search was altered as needed when searching additional databases including EMBASE, PsychINFO, CINAHL, and Cochrane.

**Figure 1:**
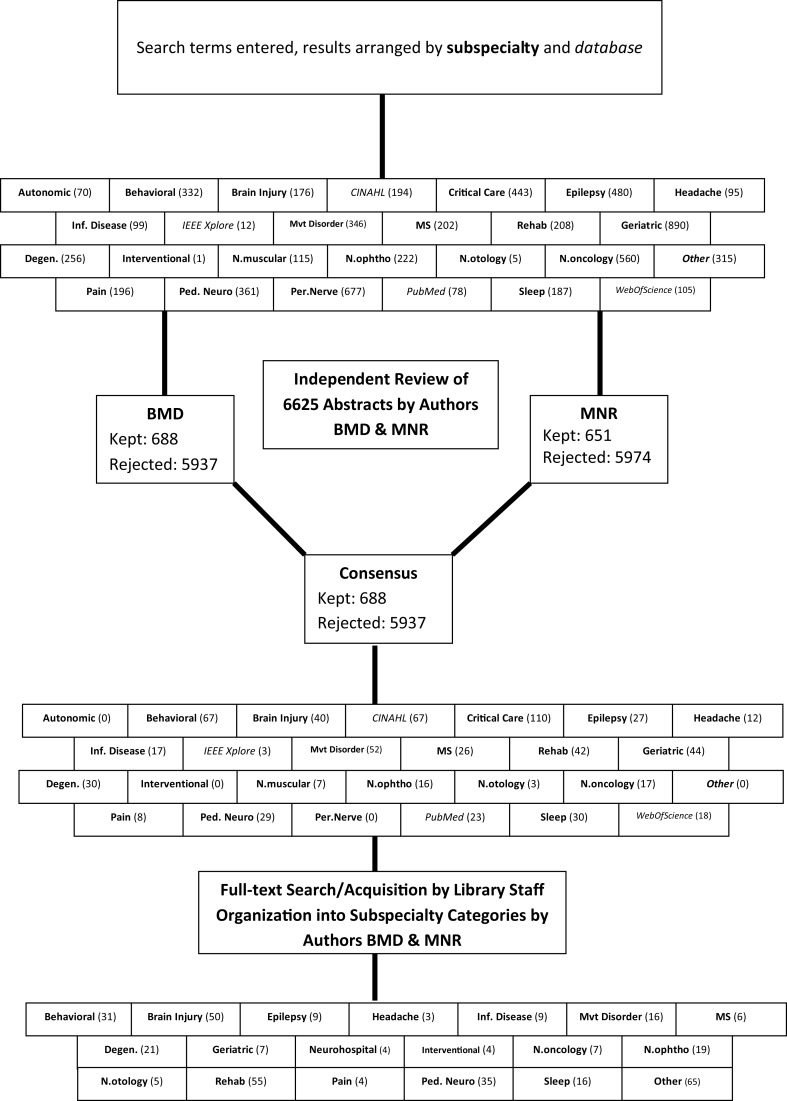
**A flowchart representing the steps of the systematic review, in order from top to bottom**. The numbers in parentheses indicate the number of search “hits” per category.

Search strategies were created in subspecialties of neurology including: autoimmune diseases, autonomic nervous system diseases, cerebrovascular disorders, critical care, epilepsy, geriatric neurology, headache, inflammatory and infectious diseases, movement disorders, multiple sclerosis, neural repair and rehabilitation, neurobehavioral diseases, neurodegenerative diseases, neuromuscular diseases, neuro-oncology, neuro-ophthalmology, neuro-otology, neurointervention, pain, pediatric and adolescent neurology, peripheral nervous system disorders, and sleep disorders. The number of results for each section, as well as a graphical depiction of the study selection process, can be seen in Figure 1.

The initial search yielded 6,625 abstracts that were independently reviewed by authors MNR and BMD. Studies that met predetermined inclusion criteria were selected for further appraisal. To be included, a study had to offer an approach to a neurologic condition using two-way AV communication. There was strong agreement in the abstract screening process (κ = 0.94) and consensus to include 688 of the studies. Of note, there were numerous “repeat hits,” where different facets of the search strategy yielded the same study, thus there were only 366 unique studies for review. The subspecialty categories were further coalesced based on available studies (as depicted in Figure 1) and a “Neurohospitalist” category was created for those studies that address the main inclusion criteria but in the inpatient setting.

**Figure 2:**
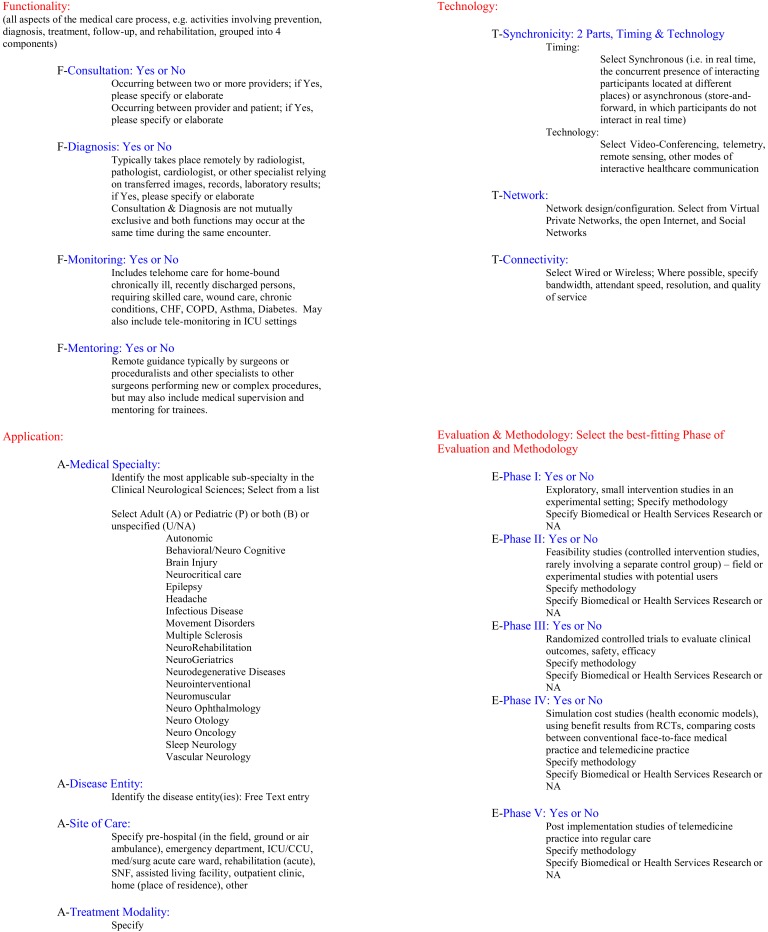
**The FATE rubric is a novel method of assessing telemedical literature for presence of salient elements including functionality, application, technology, and phase of evaluation**. Scores are assigned based on the number of “yes” answers in the “Functionality” section and the phase of evaluation.

All studies were then independently reviewed by MNR and BMD within the context of their respective subspecialty category. A rubric, inspired by Bashshur et al. (2011) and developed by BMD was adapted to score individual telemedicine studies (see Figure 2). This rubric was designed to assess and score various levels of functionality, application, technology, and evaluation phases (e.g., functionality, application, technology, and evaluative stage, FATE). Numerical scores were based on the number of “yes” answers in the functionality category (maximum of 4 points from that category) and points corresponding to the evaluation phase (maximum of 5 points from that category), for a maximum total of 9. For example, a small pilot study (1 point for evaluation phase I) focused on consultation and diagnosis (2 points from functionality category) would be expressed as FATE-3. The application and technology sections serve to succinctly summarize key elements relevant to clinical application. Once individual studies were assessed within a subspecialty category, an overall FATE score was calculated and assigned to the subspecialty. Review articles, which would otherwise have a FATE-0 score, were counted in the total number of articles scrutinized but not in the category score so as not to artificially depress an otherwise potentially mature field with many reviews.

## Discussion

To the best of our knowledge, we present the first large-scale comprehensive systematic review of teleneurological consultation studies (see Figure 1). We determined that, as hypothesized in this burgeoning field, the methodologic quality of studies is quite low. However, in a field in which large up-front costs and the “hands off” (e.g., not true face-to-face) nature of the consultation are potential if not substantial barriers to success, small-scale pilots are important and the relative dearth of high-quality randomized controlled trials, comparative efficacy studies, and health economic analyses likely reflects the nascency of teleneurologic consultation.

In addition, we offer our novel rubric (see Figure 2), inspired by the pioneering taxonomy proposed by Bashshur et al. (2011), used to assess the selected studies and arrange them by methodologic type. The FATE rubric is certainly a broad tool and may ostensibly underestimate the impact of some studies, especially smaller pilot studies. However, it serves well to acknowledge the basic parameters of a telemedical study and appropriately ranks the relatively few quality studies above others (e.g., editorials, etc.). It is not clear whether or not a higher FATE score signifies superior methodological quality of the literature, but does reflect greater depth, breadth, and maturity of telemedicine studies in a particular clinical discipline. The FATE rubric provides a tool for checking on the presence or absence of key elements of a telemedicine publication but is not a substitute for existing tools for appraisal of a studies’ methodological quality.

As the telemedical literature becomes rife with studies of stronger methodologic quality, the FATE rubric might then have a greater impact in hierarchizing the publications.

## Conflict of Interest Statement

The authors declare that the research was conducted in the absence of any commercial or financial relationship that could be construed as a potential conflict of interest.
